# Supplementary material for: Viral suppression among patients in HIV/AIDS care at healthcare facilities in Ethiopia: Same-day antiretroviral initiation
Source: PLoS One. 2025 Feb 27;20(2):e0309579. doi: 10.1371/journal.pone.0309579 (PMC11867342; doi:10.1371/journal.pone.0309579)
Supplement: Attachment — Submitted filename: PONE-D-24-34271_Response to Reviewers.docx [file pone.0309579.s001.docx]

**Response to Reviewers and editor**

We would like to express our sincere gratitude to both the reviewers and the editor for their time and support. We also acknowledge and appreciate their comments. Our special thanks to Dr. Judith Kose the academic editor for all efforts made. To clarify and address the points raised, please find our responses to each request below:

1. **Response to editor**

| Requested | Possible response |
| --- | --- |
| Financial disclosure | No change made on financial disclosure |
| If applicable, we recommend that you deposit your laboratory protocols in protocols.io to enhance the reproducibility of your results. | It is not applicable that the study didn’t involve a laboratory while it used already available lab results from the database |
| 1. Please ensure that your manuscript meets PLOS ONE's style requirements, including those for file naming. | The format was used as suggested based on the shared link. |
| 2. We note that you have indicated that there are restrictions to data sharing for this study. For studies involving human research participant data or other sensitive data, we encourage authors to share de-identified or anonymized data. However, when data cannot be publicly shared for ethical reasons, we allow authors to make their data sets available upon request. | Relevant data are within the manuscript. For further information the data that support the results of this study are available from Unisa Institutional repository: <https://uir.unisa.ac.za/handle/10500/31648>. Raw data (SPSS) can be accessed from the corresponding author upon request. |
| 3. In the online submission form, you indicated that [Insert text from online submission form here].  All PLOS journals now require all data underlying the findings described in their manuscript to be freely available to other researchers, either 1. In a public repository, 2. Within the manuscript itself, or 3. Uploaded as supplementary information. | In online submission it is indicated as data will be available from Unisa Institutional Repository while raw data can be accessed on request from corresponding author. |
| 4. Please remove your figures from within your manuscript file, leaving only the individual TIFF/EPS image files, uploaded separately. These will be automatically included in the reviewers’ PDF. | This issue resolved and done as requested. |
| 5. Please review your reference list to ensure that it is complete and correct. | References reviewed and necessary suggested corrections done. Thank you. |
| While revising your submission, please upload your figure files to the Preflight Analysis and Conversion Engine (PACE) digital diagnostic tool | I have utilized PACE to ensure that the figures comply with PLOS ONE criteria. Additionally, all pictures and tables have been removed from the manuscript. |

2. **Response to Reviewers**

| 1st Reviewer | |
| --- | --- |
| a) The authors describe VL<1000c/ml as VLS, WHO says this level results in practically zero transmission and HIV <200 as undetectable and <50/20/5 copies as viral suppression. This is not possible with this set of data as like SA- VL is defined as suppression if <1000c/ml. It is thus not current to call HIV loads<1000 as Viral suppression.- Thus the authors need to clarify this. | Thank you for your valuable comment. You are correct that, according to WHO guidelines, viral loads below 1000 copies/mL are not considered viral suppression, and viral loads under 50/20/5 copies/mL are considered undetectable. However, in Ethiopia, as of today, viral suppression is still defined as VL <1000 copies/ml. Additionally, while the management approach and protocol have evolved, with intensive follow-up now recommended for all patients with VL >50 copies/mL, the protocol during this study was limited to patients with a viral load >1000 copies/ml. |
| b) The result and conclusion sectors needs to be re written to make it more concise. | Thank you for your comment. However, the specific issues to address were not clearly indicated. Nevertheless, we have revised the results and conclusion sections based on our understanding and knowledge to make them more concise. |
| c) Only 7 patients were followed up to 24months- and thus too small a sample. The massive drop out rate is not discussed either | Thank you for your comment. This study is not a cohort study. It included patients who started treatment on the same day between October 1, 2017, and October 30, 2019. As noted in the results section, we have clarified that some patients were not eligible for viral load testing, which explains why they were not included in the specific month for testing. The viral load results are based on those who were eligible and actually underwent testing. We hope this clarification addresses your concern and provides a clearer understanding for the readers. |
| e) For a country to know what it needs to achieve to reach the WHO 95-95-95 targets for epidemic control- it needs to have data on numbers infected, numbers initiated, numbers remaining in care as well as suppression rates or numbers on treatment who have rates that will impact on transmission. Critical would be to recognise what is needed to address the challenges of retention- added counselling, intensive adherence support, community based support etc | Thank you for your comment. The aim of this study is not to evaluate the performance of the country or healthcare facilities against the WHO 95-95-95 targets. Instead, it focuses on assessing the contribution of same-day ART initiation toward achieving these targets. Specifically, our findings center on patients who started ART on the same day as part of this new initiative, providing insights into its impact on viral load suppression. The results aim to guide healthcare providers, administrators at all levels, and policymakers in understanding the implementation and evidence-based outcomes of same-day ART initiation within the Ethiopian healthcare system. |
| 2nd reviewer | |
| a) The manuscript is technically sound, methodology is clear, and data is presented well. The findings follow logically from the hypothesis to the conclusion. The data collected is in line with the objectives of the study and supports the authors’ claims.   **Reviosn required**  The reference list requires attention, specifically reference number 3, which cites the World Health Organization (WHO). It is important to ensure that the name is formatted correctly and consistently throughout the manuscript. Please revise this entry to accurately reflect the proper citation format for WHO. | Thank you so much. The identified gaps have been addressed, and we have ensured that all references are included as indicated. |

Thank you

End!
